# RB1 Status in Triple Negative Breast Cancer Cells Dictates Response to Radiation Treatment and Selective Therapeutic Drugs

**DOI:** 10.1371/journal.pone.0078641

**Published:** 2013-11-12

**Authors:** Tyler J. W. Robinson, Jeff C. Liu, Frederick Vizeacoumar, Thomas Sun, Neil Maclean, Sean E. Egan, Aaron D. Schimmer, Alessandro Datti, Eldad Zacksenhaus

**Affiliations:** 1 Department of Laboratory Medicine and Pathobiology, University of Toronto, Toronto, Ontario, Canada; 2 Division of Advanced Diagnostics, Toronto General Research Institute, University Health Network, Toronto, Ontario, Canada; 3 S.M.A.R.T. High Throughput Facility, Mount Sinai Hospital, Samuel Lunenfeld Research Institute, Toronto, Ontario, Canada; 4 Clinical Studies Resource Centre, OCI, University Health Network, Toronto, Ontario, Canada; 5 Program in Developmental and Stem Cell Biology, Hospital for Sick Children, Molecular Genetics, University of Toronto, Toronto, Ontario, Canada; 6 Department of Experimental Medicine and Biochemical Sciences, University of Perugia, Perugia, Italy; Wayne State University School of Medicine, United States of America

## Abstract

Triple negative breast cancer (TNBC) includes basal-like and claudin-low subtypes for which only chemotherapy and radiation therapy are currently available. The retinoblastoma (RB1) tumor suppressor is frequently lost in human TNBC. Knockdown of RB1 in luminal BC cells was shown to affect response to endocrine, radiation and several antineoplastic drugs. However, the effect of RB1 status on radiation and chemo-sensitivity in TNBC cells and whether RB1 status affects response to divergent or specific treatment are unknown. Using multiple basal-like and claudin-low cell lines, we hereby demonstrate that RB-negative TNBC cell lines are highly sensitive to gamma-irradiation, and moderately more sensitive to doxorubicin and methotrexate compared to RB-positive TNBC cell lines. In contrast, RB1 status did not affect sensitivity of TNBC cells to multiple other drugs including cisplatin (CDDP), 5-fluorouracil, idarubicin, epirubicin, PRIMA-1^met^, fludarabine and PD-0332991, some of which are used to treat TNBC patients. Moreover, a non-biased screen of ∼3400 compounds, including FDA-approved drugs, revealed similar sensitivity of RB-proficient and -deficient TNBC cells. Finally, ESA^+^/CD24^−/low^/CD44^+^ cancer stem cells from RB-negative TNBC lines were consistently more sensitive to gamma-irradiation than RB-positive lines, whereas the effect of chemotherapy on the cancer stem cell fraction varied irrespective of RB1 expression. Our results suggest that patients carrying RB-deficient TNBCs would benefit from gamma-irradiation as well as doxorubicin and methotrexate therapy, but not necessarily from many other anti-neoplastic drugs.

## Introduction

Triple negative breast cancer (TNBC) represents a collection of tumors that lack expression of estrogen (ER) and progesterone (PR) receptors as well as the receptor tyrosine kinase HER2 [1]. These tumors can be further subdivided into basal-like, claudin-low and other subclasses. The former is characterized by expression of basal markers and elevated proliferation. The claudin-low subtype lacks basal markers but expresses low levels of tight junction proteins and cell adhesion proteins such as E-cadherin and certain claudins, as well as high levels of genes associated with epithelial-mesenchymal-transition (EMT) [2], [3].

TNBC makes up 10–30% of all breast cancer cases. Compared to other subtypes, TN tumors are associated with poor prognosis, in part due to a lack of targeted treatment. Clinically, TNBCs respond more favorably to chemotherapy than other types, however prognosis still remains poor due to a greater risk of distal recurrence, with a rapid rise in relapse in the first 3 years post diagnosis [4]–[6]. Metastatic disease is extremely aggressive, and often arises in tissues that are difficult to treat, such as bone or brain. Therefore, it is pertinent to find more effective treatments for aggressive forms of TNBC.

The tumor suppressor RB1 is often lost by mutation, deletion or transcriptional silencing as well as by hyper-phosphorylation of its gene product, pRb, in many human malignancies [7]–[9]. Indeed, it is deleted or rearranged in ∼20–25% of BC cell lines [10]–[18]. It is primarily inactivated in TNBC [19]. Furthermore, recent genomic sequencing, transcriptome analysis, epigenetic and proteomic analysis identified RB1 loss in ∼20% of TNBC [20]. Deletion of murine Rb in mammary epithelium induces basal-like and luminal tumors, whereas deletion of both Rb and p53 leads to claudin-low like tumors [21], hence demonstrating a causal role for RB1 in TNBC.

Acute inactivation of RB1 in hormone-dependent luminal breast and colon cancer cells increases response to several antineoplastic drugs, suggesting that RB-deficiency affects therapeutic outcome in certain tumor types including ER^+^ breast cancer. However, RB1 is most commonly lost in TNBC, not in ER^+^ luminal tumors [20], and therefore it is important to determine the effect of RB1 status in TNBC lines on response to therapy. Moreover, whether this effect is due to acute inactivation of RB1 and whether it can be seen in RB1-mutant TNBC is not known. Moreover, whether RB status has a general effect on chemo-sensitivity to multiple drugs has not been addressed. Finally, it is not clear whether improved clinical outcome of patients carrying RB-deficient tumors is due to better response to chemotherapy or better response to irradiation. Here, we determined the effect of RB1 status on sensitivity of TNBC cells as well as the cancer stem cell (CSC) fraction to gamma-irradiation and multiple anti-neoplastic drugs. Surprisingly, we found that RB1 status affects response to irradiation and doxorubicin, which are used to treat invasive TNBC, but not to most other anti-neoplastic drugs commonly used to treat TNBC and other BC subtypes. Moreover, only radiation affected the CSC fraction from RB-deficient TNBC lines more than from RB-proficient TNBC cells.

## Results

### pRb protein is lost in ∼30% of basal-like and claudin-low TNBC cell lines

**Figure 1:**
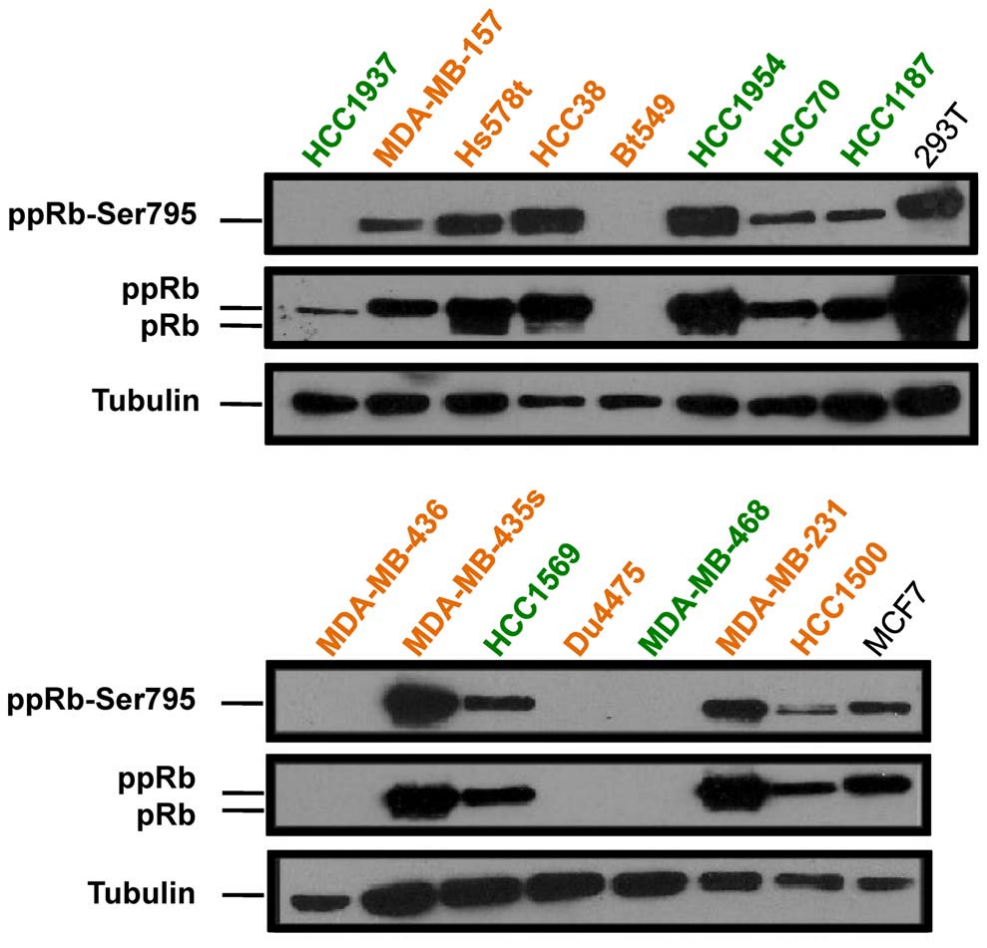
Status of pRb protein in human triple negative breast cancer (TNBC) cell lines. Western blot analysis of pRb and phospho-ppRb-Ser795 in TNBC derived lines. HCC1937 expresses mutant pRb. MDA-MB-231, MDA-MB-435, MDA-MB-436, MDA-MB-157, Bt549, Du4475, Hs578T and HCC38 are claudin-low (BaB – depicted in orange). HCC70, HCC1937, HCC1954, HCC1187, HCC1569 and MDA-MB-468 are basal-like (BaA – depicted in green). MCF7, a luminal BC line, and 293T, a transformed kidney epithelium line, were used as control. Tubulin served as a loading control.

BC cell lines were shown to maintain many genomic and transcriptional characteristics of primary breast tumors from which they were derived and are therefore useful as surrogates for breast cancer [12]. TNBC cell lines were established for basal-like (Basal-A) and mesenchymal/claudin-low (Basal-B) tumors. To analyze the effect of RB1 status on the response of TNBC cells to chemotherapy, we first determined its expression and phosphorylation status in a panel of 15 TNBC cell lines, which included 6 basal-like and 9 claudin-low lines (**Figure 1A**). Of the 6 basal-like lines, one (MDA-MB-468) exhibited no pRb expression, whereas another (HCC1937) exhibited low level of normal size pRb that was not phosphorylated, indicative of a small in-frame deletion. Among the 9 claudin-low lines, three (Bt549, Du4475 and MDA-MB-436) were completely devoid of RB1 expression. Thus, in both TNBC subtypes, wild-type pRb was absent in 33% of cases (2/6 and 3/9, respectively). Within RB-positive lines there was a degree of variability in the level of phosphorylation, as determined using anti-phospho-Ser795-pRb antibody. In 5 of these (HCC1187, MDA-MB-157, HCC70, HCC1569, and HCC1500), pRb was almost completely phosphorylated (hyper-phosphorylated), depicted by a single, slow-migrating band when probed for total pRb. The remaining 5 lines (Hs578t, HCC1954, HCC38, MDA-MB-435, and MDA-MB-231) displayed a doublet band, representing both hyper- (upper band) and hypo- (lower band) phosphorylated forms of pRb. Taken together, 66% (10/15) of the lines had functionally inactive pRb (5 RB1 null and 5 pRb hyper-phosphorylated), whereas the remaining five lines showed cell cycle regulated phosphorylation seen in normal cells with an intact pRb pathway.

### Microarray analysis of the RB1 pathway in TNBC lines

**Figure 2:**
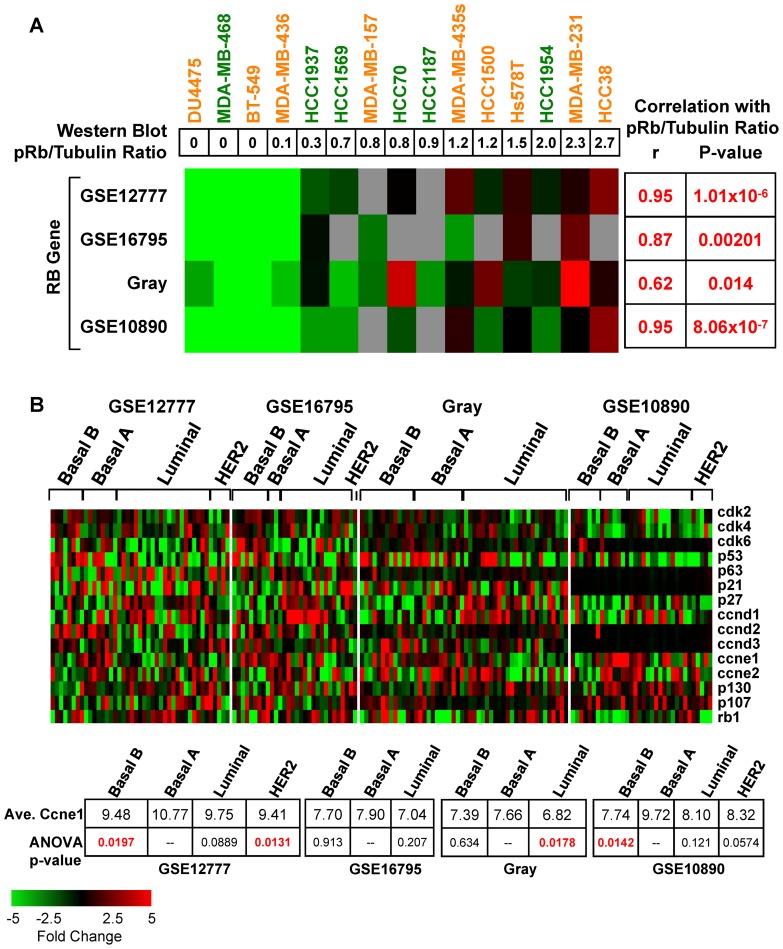
Status of RB1 gene in human triple negative breast cancer (TNBC) cell lines. (**A**) RB1 RNA expression correlates with pRb protein expression. Top, ratio of pRb to Tubulin from Figure 1. Botton, RNA expression of RB1 in indicated cell lines in 4 different studies and Pearson's correlation (r) relative to pRb protein expression. (**B**) Microarray analysis of cyclins and Cdks on the RB-pathway in human derived breast cancer cell lines, clustered according to subtype, from the 4 data sets. Cyclin E1 was consistently elevated in basal-A but not in basal-B tumors.

The aforementioned Western blot analysis allowed us to compare pRb protein expression to RB1 transcript levels using publicly available microarray data sets for over 20 TNBC cell lines. We used the following data sets: Neve/Gray [12], GSE12777 [22], GSE16795 [23], and GSE10890 (deposited by Genentech). We calculated the correlation of pRb protein level (average pRb/tubulin expression) with RB1 RNA abundance in the 4 different microarray data sets (**Figure 2A**). This analysis revealed very high correlation between pRb protein and RNA expression (average of 4 cohorts  = 0.85, p<0.01), suggesting that RB1 RNA levels may be used as surrogate for pRb protein expression.

We next investigated the expression RB1 relative to components of the RB1 pathway including the cyclins (D1, D2, D3, E1, and E2) and cyclin-dependent kinases (Cdk4, Cdk6 and Cdk2), using the same four published microarray data sets (**Figure 2B**). The panels were clustered based on subtype (basal-like/Basal A, claudin-low/Basal B, luminal and HER2^+^ breast cancer). Consistent with a previous observation that cyclin E1 is elevated in basal-like breast cancer [20], we found that cyclin E1 was also elevated in basal-A tumors compared to all other types (**Figure 2B**). Interestingly, cyclin E1 was elevated in basal-A (basal-like) but not in basal-B (claudin-low) TNBC lines. No other correlation between cyclins/Cdks expression was evident in basal-A vs. basal-B, or RB^+^ vs. RB^−^ TNBC lines.

### RB1 deficiency sensitizes TNBC cells to radiation therapy, doxorubicin and methotrexate but not to the CDK4/6 inhibitor PD-0332991, CDDP or 5-fluorouracil

**Figure 3:**
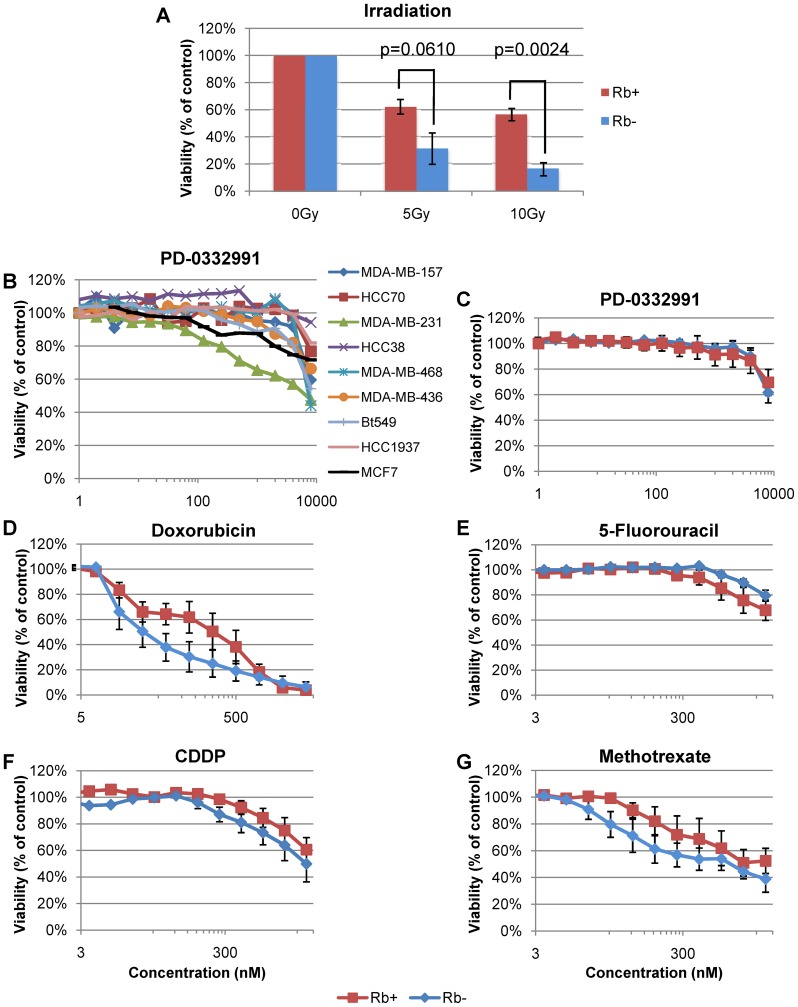
Effect of RB1 status on response to radiation and anti-neoplastic drugs of TNBC lines. Treatment with (**A**) gamma-irradiation, (**B**–**C**) CDK4/6 inhibitor PD-0332991, (**D**) doxorubicin, (**E**) 5-fluorouracil, (**F**) CDDP, and (**G**) methotrexate. RB^+^ lines: MDA-MB-231, HCC38, Hs578t and HCC70. RB^−^ lines: MDA-MB-468, MDA-MB-436, Bt549 and Du4475. Values represent the average of 3–4 assays, each performed in triplicate. p-values for gamma-irradiation by two-tailed t-test: 5 Gy, p = 0.0610; 10 Gy, p = 0.0024. p-values for curve sets calculated using nonlinear regression analysis: doxorubicin, p = 0.0140; CDDP, p = 0.0515; 5-fluorouracil, p = 0.0187; methotrexate, p = 0.0043.

In addition to chemotherapy, pre- and post-operative radiation is often applied locally after excision of invasive BC tumors [24]–[27]. To investigate the effects of RB1 status on sensitivity to gamma-irradiation, we performed MTT viability assays on 8 cell lines (4 RB^+^, 4 RB^-^) treated with 5–10 Gy. The RB-null lines analyzed were MDA-MB-468, Du4475, MDA436 and Bt549; RB-proficient lines were MDA-MB-231, HCC38, HCC70 and Hs578t. Interestingly, although radiation treatment was effective against all TNBC lines, the RB-null lines were significantly more sensitive to radiation than RB-positive lines at a dose of 10 Gy (p = 0.0024) (**Figure 3A**).

We next assessed the efficacy of a new small molecule inhibitor of cyclin 4/6, PD-0332991, which is being evaluated in clinical trials for several cancer types [28], [29]. A recent study showed that luminal but not basal-like breast tumors are highly sensitive to this drug [30]. We therefore asked whether TNBC cells with highly phosphorylated pRb are more sensitive to this drug than RB-deficient, or RB-proficient lines with low pRb phosphorylation. Surprisingly, we found that PD-0332991 only decreased viability of one RB^+^ TNBC line, MDA-MB-231, with an IC_50_ of 4 µM, whereas all other lines were highly and equally resistant to PD-0332991 (**Figure 3B**–**C**). As expected, the control luminal MCF7 breast cancer line was more sensitive than the TNBC lines. Thus, RB1 status in TNBC cells affects response to radiation, but not to CDK4/6 inhibition via PD-0332991.

Next, we investigated the effect of RB1 status on TNBC cell response to doxorubicin, methotrexate, cisplatin (CDDP) and 5-fluorouracil (5-FU), which are commonly used to treat TNBC [31]. Interestingly, the RB-null lines were more sensitive to doxorubicin (p = 0.0140) and methotrexate (p = 0.0043). In contrast, the RB-proficient lines were more sensitive to 5-FU (p = 0.0187), and there was no statistically different response of these lines to CDDP (**Figure 3D**–**G**).

### High throughput library screen reveals similar sensitivity of RB^−^ versus RB^+^ TNBC tumor cells

**Figure 4:**
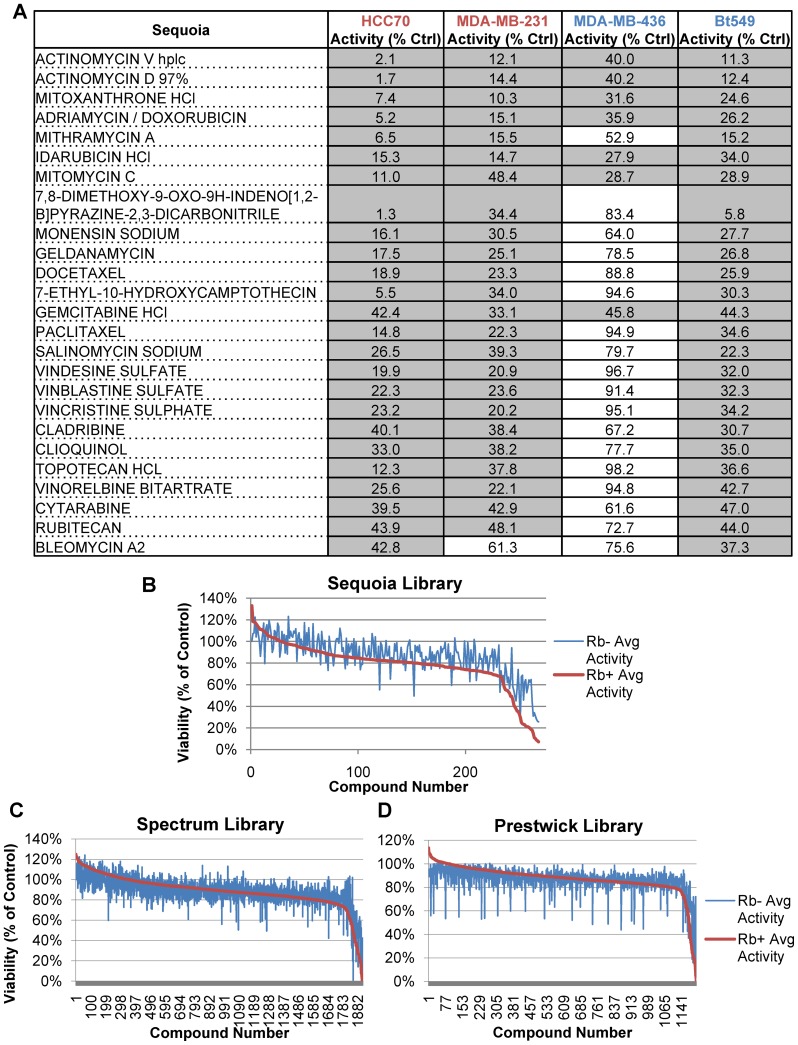
Similar drug sensitivity observed following high-throughput screen of ∼3400 drugs against human RB^+^ vs. RB^−^ TNBC lines. RB^+^ lines (red): HCC70 and MDA-MB-231. RB^−^ lines (blue): MDA-MB-436 and Bt549. (**A**) Top 25 hits from Sequoia library (1 µM, 268 drugs) ranked by average efficacy. Shaded values represent viability ≤50%. (**B–D**) Sensitivity of RB^−^ cells relative to RB^+^ cells following screens with the Sequoia, Spectrum (1 µM, 2000 drugs) and Prestwick (0.8 µM, 1185 drugs) libraries. Arrow points to phenylmercuric acetate (see text).

To determine whether RB1 status had a global effect on chemo-sensitivity, we performed a screen of 268 FDA-approved drugs using two RB^−^ (MDA-MB-436, Bt549) and 2 RB^+^ TNBC lines (HCC70, MDA-MB-231). The majority of strong hits were known chemotherapeutics such as doxorubicin and idarubicin (**Figure 4A**). Sensitivity of the RB-negative lines to each drug in comparison to the RB-proficient lines is plotted in **Figure 4B**. While small fluctuations in response were observed, there was no general increase in sensitivity of the RB-mutant lines. In fact, on average, the RB deficient lines were slightly more resistant than RB proficient lines. Indeed, when we plotted drug sensitivity ranked by RB1 status and superimposed the curves, we found that RB^+^ tumors were more sensitive than RB^−^ tumor cells to any of the FDA approved drugs (**Figure S1**). In another study we screened 3185 compounds against the same 4 TNBC lines [32]. When compared based on RB1 status, we again found a slight increase or no difference in response between RB proficient and RB deficient lines (**Figure 4C**–**D** **; Figure S1).** Thus, loss of RB1 does not lead to a general increase in chemo-sensitivity to a multitude of compounds, including antineoplastic drugs. Notably, one drug, phenylmercuric acetate, diminished growth of the RB^−^ lines to 1% viability (Figure 4C, arrow). This same drug also reduced viability of one RB^+^ line, HCC70, to 1%, but the other RB^+^ line, MDA-MB-231, was completely resistant (96% viability), therefore leading to an average of 48% inhibition. Given these results, and the fact that phenylmercuric acetate is a mercury-containing compound [33], [34], we did not further pursue this drug.

**Figure 5:**
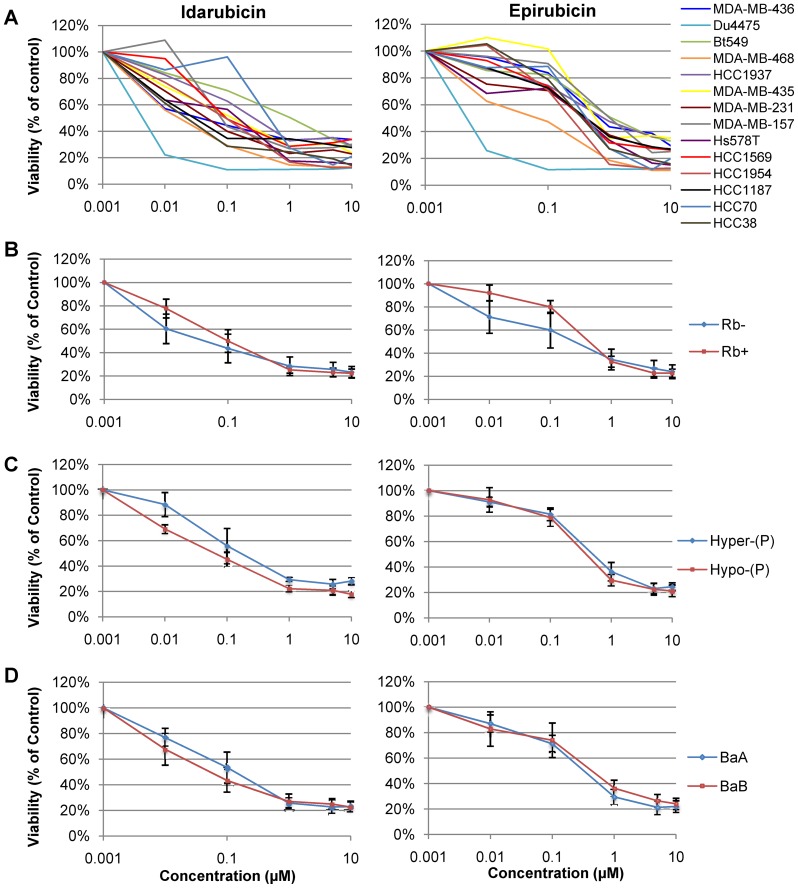
Effect of RB1 status on response of 14 human derived TNBC lines to idarubicin or epirubicin. (**A**) Response of each individual line. Values represent the average of 3–5 assays, each performed in triplicate. (**B**) Average response based on RB1 status. RB^+^ lines: MDA-MB-231, HCC38, Hs578t, MDA-MB-157, HCC1954, HCC1569, HCC3153, SUM149 and HCC70. RB^−^ lines: MDA-MB-436, MDA-MB-468, Bt549, Du4475 and HCC1937. Idarubicin, p = 0.3837; epirubicin, p = 0.1083. (**C**) Average response for hyper- and hypo-phosphorylated pRb states. epirubicin, p = 0.7905; idarubicin, p = 0.0353. (**D**) Average response for basal A (BaA) and basal B (BaB) TNBC subtypes. epirubicin, p = 0.6579. idarubicin, p = 0.5775. p-values calculated using nonlinear regression analysis.

Next, we analyzed the effect of RB1 status on selected anti-neoplastic drugs used to treat different types and grades of breast cancer. For this, we used our panel of 9 RB^+^ and 5 RB^−^ lines, representing basal-like and claudin-low subtypes. Drugs tested included idarubicin, which scored highly in our screen of TNBC lines (**Figure 4A**), and epirubicin as well as fludarabine [31], [35]–[38]. Notably, idarubicin is used to treat hormone-resistant, metastatic TNBC patients [39]–[41]. We also tested the effect of PRIMA-1^met^, a small molecule that reactivates and restores function to mutant p53 [42]. We found varied response to each drug (**Figure 5A**, **Figure S2A**). However, there was no significant difference between the average dose response curves for RB^+^ and RB^−^ lines (**Figure 5B**; **Figure S2B**). pRb phosphorylation state did not affect response to treatment with these drugs, with the exception of idarubicin, which contrary to our expectations killed hypo-phosphorylated pRb lines slightly better than hyper-phosphorylated pRb cells (p = 0.035; **Figure 5C**, **Figure S2C**). There was also no difference in response to these chemotherapeutics between basal-like (Basal A) and claudin-low (Basal B) cell lines (**Figure 5D**, **Figure S2D**). We note that two of the TNBC lines we analyzed (MDA-MB-436, HCC1937) carry both RB1 and BRCA1 mutations. Drug sensitivity of these tumor cells was indistinguishable from sensitivity of other RB**^−^** (but Brca1 proficient) or RB^+^ TNBC cell lines (data not shown).

### RB1 status affects sensitivity of triple negative cancer stem cell (CSC) fraction to irradiation but not chemotherapy

**Figure 6:**
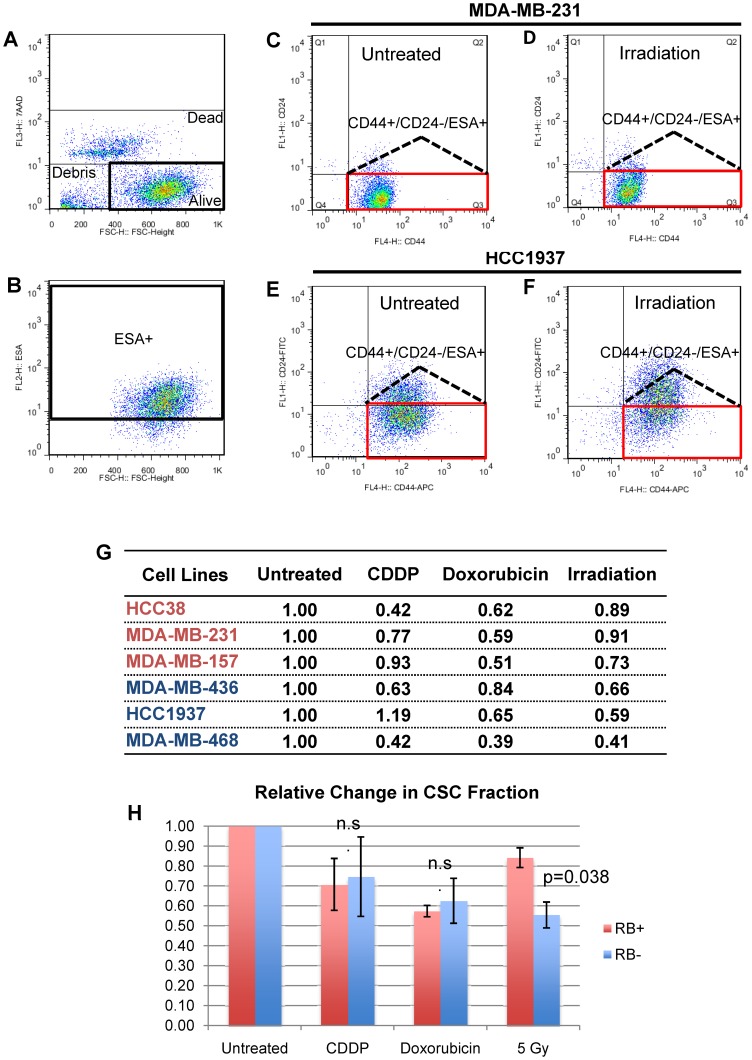
Effect of RB1 status on response to radiation or doxorubicin of CD44^+^/CD24^−/low^/ESA^+^ cancer stem cell (CSC)-enriched fraction in human TNBC lines. Treatment with 5 Gy radiation or IC_50_ doses of CDDP or doxorubicin. (**A**) Viable cell population of 7-AAD control MDA-MB-231 cells. (**B**) ESA positive cell fraction of untreated MDA-MB-231 cells. (**C–D**) MDA-MB-231 CSC fraction in untreated and irradiated cells. (**E–F**) HCC1937 CSC fraction in untreated and irradiated cells. (**G**) Relative change in the CSC fraction for each line treated with CDDP, doxorubicin or radiation. (**H**) Average relative ratio of the CSC fraction for RB^+^ and RB^−^ TNBC cell lines treated as indicated. RB^+^ lines (red): MDA-MB-231, MDA-MB-157 and HCC38. RB^−^ lines (blue): MDA-MB-436, MDA-MB-468, and HCC1937. p-value calculated using a two-tailed t-test. n.s.  =  not significant.

There is strong evidence that divergent types of cancers, including those of the breast, are organized in a hierarchy with cancer stem cells (CSCs), capable of sustaining tumorigenesis, at their apex [43], [44]. As CSCs and their non-CSC derivatives exhibit distinct sensitivity to therapy [45]–[48], we asked whether RB1 status could affect response of CSCs to anti-neoplastic drugs or gamma-irradiation. CSCs can be functionally identified on the basis of cell surface markers by flow cytometry analysis and transplantation experiments. Specifically, in TNBC cell lines CSCs were identified as 7AAD^−^/ESA^+^/CD24^−/low^/CD44^+^ [49]. To determine the effects of chemotherapy and irradiation on the CSC fraction of TNBC lines, we treated RB^+^ and RB^−^ lines with CDDP, doxorubicin or gamma-irradiation (**Figure 6**). All three treatments were found to reduce the relative ESA^+^/CD24^−/low^/CD44^+^ CSC fraction in each line (**Figure 6G**). Importantly, treatment with as little as 5 Gy significantly inhibited the CSC fraction in RB^−^ TNBC cells compared to RB^+^ lines (**Figure 6H**, p = 0.038). In contrast, RB1 status did not affect response of CSCs to CDDP or doxorubicin. We conclude that RB-deficiency increases response to gamma-irradiation at least in part by enhancing the killing of TNBC CSCs, but has either moderate (e.g. doxorubicin) or negligible (most anti-neoplastic drugs) effect on sensitivity of CSCs to chemotherapy.

## Discussion

### RB1 status in TNBC

We found that pRb expression is lost in 33% of human basal-like cell lines, in agreement with recent genomic analysis of primary basal-like tumors [50]–[53]. We also found that pRb protein expression is lost in 33% of human claudin-low TNBC lines. Interestingly, RB1 mRNA expression significantly correlated with pRb protein expression, suggesting that transcript levels may serve as surrogate for pRb protein. However, in one study, it was reported that pRb protein expression detected by immunohistochemistry is lost in nearly most human TNBC samples [54], and therefore similar analysis (comparing RB1 expression by RNA to pRb expression by immunostaining) is required to establish this correlation in tumor samples. We showed that pRb was heavily phosphorylated, hence inactivated, in many RB1-proficient TNBC tumor lines, and this correlated with high expression of Cyclin E1 in basal-A but not in basal-B tumors. Possibly, post-transcriptional activation of the CDKs or cooperative effect of several cyclins/Cdks drives pRb phosphorylation in basal-B cells. All together, pRb is either genetically or functionally inactivated in 66% of TNBC cell lines tested. However, in this study, we only tested the effect of pRb status (presence or absence) or response to therapy.

### Role of RB1 during therapeutic challenge

Inactivation of RB1 is thought to increase sensitivity to DNA damaging agents, as cells are unable to halt cell cycle progression and repair damaged DNA. This notion is supported by studies on BC patient cohorts showing that women with triple negative disease have a better prognosis and longer disease-free-survival if they are pRb-deficient and treated with chemotherapy [55]–[57]. However, patients with invasive breast cancer often receive both local radiation and systemic chemotherapy [58], [59], and these studies did not clearly differentiate between women who received chemotherapy alone or chemotherapy plus radiotherapy. Our analysis of TNBC cell lines demonstrates that RB null lines are significantly more sensitive to radiation treatment than RB proficient cells, whereas response to chemotherapy is variable. Our results therefore raise the possibility that the improved survival of RB-negative TNBC patients may be due to irradiation and doxorubicin or perhaps a combination of both, but not due to most other chemotherapies.

Previous studies using hormone-sensitive RB1 proficient BC lines (MCF7, Zr-75-1, and T47D) demonstrated that silencing of RB1 significantly increased sensitivity to 5-FU, methotrexate and CDDP [51], [55]. It is possible that this increased sensitivity was due to the effect of acute inactivation of RB1, whereas established RB1 null cancer cells, used here, have adapted other mechanisms for DNA repair and apoptosis evasion, or have rewired to compensate for RB loss through activation of p107 [60].

Another recent study shows that an RB-pathway-loss signature predicts response of ER^+^ and basal-like breast cancer patients to therapy [61]. Increased response may be due to treatments with drugs/radiation to which RB-deficient tumors are indeed more sensitive as shown here. Alternatively, the RB-pathway loss signature may identify highly proliferating cells, not necessarily RB-deficient cells, and it is the high proliferation of these tumors that may underlie increased sensitivity to chemotherapy. Indeed, the frequency of RB-pathway signature-positive tumors far exceeds the frequency of RB-deficiency, especially in the ER^+^ cohorts. It would be important to determine whether TNBC cells with RB-pathway disruption are as sensitive to irradiation and doxorubicin as *bona fide* RB-deficient TNBC cells, and whether they share a similar spectrum of drug sensitivity.

Our study demonstrates no difference in sensitivity between RB-deficient and RB-proficient cell lines towards a large number of anti-neoplastic drugs and other compounds. However, we identified selective response to specific compounds. For example, methotrexate inhibited RB^−^ lines by 10–20% more than RB^+^ lines along the entire dose-response curve (p = 0.0043). Methotrexate exerts its effect by inhibiting purine/DNA synthesis. Therefore, one would expect that other drugs affecting DNA synthesis would also be more effective against RB-deficient TNBC cells. However, this is clearly not the case since treatment with 5-FU, which inhibits thymidine synthesis, shows a reversed sensitivity profile with RB^−^ cells being less sensitive than RB^+^ lines (Fig. 3). Furthermore, epirubicin and doxorubicin are both intercalating agents, however only doxorubicin was found to target RB^−^ lines more efficiently than RB^+^ cells (p = 0.0140). Finally, CDDP exerts its effect by cross-linking DNA, where RB-independent pathways regulate response to this stress [62]. One might expect RB1 loss to potentiate the response to CDDP, as was seen with acute RB1 knockdown [51], [55]. However, we found that CDDP did not have a statistically significant effect on RB^−^ cells relative to RB^+^ TNBC lines. Together, these results suggest that RB status affects response to specific drugs, and this may be important for development of drugs that selectively target RB-deficient tumor cells.

Intriguingly, our findings on the role of RB1 status suggest that radiation therapy is most effective against TNBC lacking pRb protein. We observed a decrease in cell viability for RB-null lines when treated with 5 Gy of radiation, and a further decrease at 10 Gy (p = 0.0024). Moreover, the ESA^+^/CD24^−/low^/CD44^+^ CSC fraction in RB-null TNBC lines was significantly more susceptible to as low doses of irradiation (5 Gy) compared to CSCs from RB-proficient lines (p = 0.038). In the clinic, TNBC patients may receive a biological equivalence of >60 Gy [63]–[65], and we therefore expect the difference between RB-deficient and -proficient tumor cells to be even greater at higher doses. Breast cancer treatment regimes are becoming more refined and tailored toward specific cancer subtypes, and ultimately toward each specific tumor. Our results indicate that identification of RB1 status may guide radiation and specific drug therapies. Specifically, patients with RB-deficient TNBC may benefit from radiotherapy in combination with doxorubicin.

## Materials and Methods

### Cell lines and Cultures

MDA-MB-231, MDA-MB-468, MDA-MB-157, Hs578T, and MCF7 were maintained in DMEM containing 10% FBS and 1% PEST. MDA-MB-436 was maintained in DMEM containing 10% FBS, 1% PEST, and supplemented with 10 µg/ml insulin. HCC70, HCC1937, HCC38, HCC1954, HCC1569, HCC1187, HCC3153, HCC1500, Du4475, and Bt549 were maintained in RPMI containing 10% FBS and 1% PEST. All cell lines were grown at 37°C with 5% CO_2_ in attachment plates (Du4475 grew in attachment plates as suspended aggregates). Cell lines Du4475, Bt549, MDA-MB-436, MDA-MB-468, MDA-MB-231, and HCC1569 were kind gifts from Dr. Mona Gauthier. HCC3153 was a gift from Dr. Tak Wah Mak lab. The remaining breast cancer cell lines were purchased from the America Type Culture Collection (ATCC) (Manassas, VA, USA). All cell lines are available from the ATCC.

### Western Blotting

Each TNBC cell line was cultured in 10 cm dishes with their corresponding media, treated with 0.25% trypsin (Sigma-Aldrich), washed with PBS, pelleted and lysed with lysis buffer (0.15 M NaCl, 1% Tritonx100, 5 mM EDTA, 5 mM NaF, 0.5 mM Na_3_VO_4_, and 1∶100 protease inhibitor cocktail [1 mg/mL leupeptin, 2 µg/mL aprotinin, and 100 mM PMSF]). Protein concentration was determined by Bio-Rad dye-binding assay (Bio-Rad, Hercules, CA). Proteins in total cell lysates were fractionated by SDS-PAGE and transferred onto nitrocellulose membranes using electrophoresis for subsequent immunoblotting. Membranes were blocked with 5% nonfat dried milk in phosphate-buffered saline containing 0.05% Tween 20 (PBST) at R.T. for 1 h and incubated at 4°C overnight with mouse anti-pRb primary antibody (Cell Signaling), rabbit anti-ppRb-ser795 primary antibody (Cell Signaling), or rabbit anti-tubulin primary antibody (Cell Signaling). Membranes were washed with PBST buffer 3 times, 5 min each and incubated with HRP-conjugated anti-mouse, or anti-rabbit, IgG secondary antibody (Cell Signaling) for 1 h. After further washing, the membranes were allowed to react with ECL (enhanced chemiluminescence substrate, Thermo Scientific), the signal was detected using autoradiography film and developed using a Konica SRX-101A developer. Primary antibodies were diluted 1∶1000 in PBS with 5% BSA; secondary antibodies were diluted 1∶2000 in PBS with 5% nonfat dried milk.

### Drug Screening

Screens were performed in the S.M.A.R.T. Facility of the Samuel Lunenfeld Research Institute. All libraries were prepared in 100% DMSO to facilitate pinning. The final concentration of DMSO in each screen was 0.4%. The breast cancer lines MDA-MB-231, MDA-MB-436, Bt549, and HCC70 were seeded with their corresponding media in 384-well plates at a density of 900 cells/well in a total volume of 50 µL/well. The following day, plates were pinned with a drug library to reach a final concentration of 1 µM (Sequoia and Spectrum libraries), or 0.8 µM (Prestwick library). Alamar blue (Invitrogen) was added three days post drug pinning at 10% of the volume (5 µL/well), and cell viability was read 4–6 h later using a Pherastar plate reader. The Spectrum library consists of 2000 drugs from MicroSource Discovery Systems (USA) (http://www.msdiscovery.com/spectrum.html), which includes marketed drugs, natural products with unknown biological properties, and other non-drug molecular entities. The Prestwick library is composed of 1185 drugs from Prestwick Chemical (Illkirch, France) (http://www.prestwickchemical.com/index.php?pa=26), and contains only marketed drugs approved by the FDA, EMEA and other agencies. Both libraries have been successfully implemented in drug reposition and combination strategies [66], [67]. Screen data were normalized using the B-score approach to select statistically relevant hits after correction for positional effects and general systematic errors during incubation [68].

### Drugs and Irradiation

Unless otherwise stated, drugs used in this study were obtained from Sigma-Aldrich (Oakville, Ontario, Canada). Compounds used to establish dose-response curves include cisplatin/CDDP, doxorubicin, idarubicin, epirubicin, 5-fluorouracil, methotrexate, fludarabine, PRIMA-1^met^ (Tocris Bioscience, Minneapolis, MN, USA), and PD-0332991 (Selleckchem, Houston, TX, USA). The source of radiation was from a Cesium-137 Gammacell Irradiator.

### MTT Viability Assays

Cells were seeded in 96-well plates at their optimal density (2–5×10^3^ cells/well) and treated the following day, leaving 100 µL final volume of media. Three days (72 h) post treatment 30 µL of 2 mg/mL MTT (3-(4,5-dimethylthiazol-2-yl)-2, 5-diphenyl tetrazolium bromide, Sigma-Aldrich) was added to each well and incubated for 2–4 h, depending on cell type. MTT/media solution was aspirated and replaced with 100 µL DMSO and left at R.T. for 15–20 min to dissolve the formazan dye. After gentle agitation to ensure even mixture of the dye, a 96-well microplate reader (Molecular Devices) was used to determine the optical density (OD) of each well at 570 nm. Viability (%) was determined by (treatment group OD/untreated control group OD) ×100%, using DMSO as a blank. Each assay was performed in triplicate, and repeated at least 3 times.

### Flow Cytometry

Cell lines were plated at optimal densities and treated the following day with their respective IC50 of cisplatin/CDDP or doxorubicin, or with exposure to cesium-137 radiation to a dose of 5 Gy. After 72 h, the supernatant from each treatment group was collected before cells were trypsinized to single cell suspension. Trypsinized cells were added to their respective supernatants, washed with PBS, pelleted, resuspended in serum-free PBS, and counted. In a volume of 100 µL serum-free PBS, 0.5–1.0×10^6^ cells were incubated with 5 µL mouse anti-human ESA/Ep-CAM/CD326-PE (BioLegend), CD24-FITC (BD Biosciences), and CD44-APC (BD Biosciences) antibody at R.T., in the dark, for 15–20 min with occasional pulse vortexing. Cells were then washed and strained to single cells into 5 mL polystyrene round-bottom FACS tubes (BD Falcon) to a total volume of 500 µL (1×10^6^ cells/mL). Finally, 5 µL 7-AAD (BD Biosciences) was added to each tube as a viability marker, and cells were processed on a FACSCaliber (Becton Dickinson) no longer than 1 h post-staining. In all experiments, 7-AAD exclusion, side scatter and forward scatter profiles were used to eliminate dead cells and debris.

### Statistical Methods

Statistical analyses for curve comparisons were performed on Prism 6 GraphPad Software using nonlinear regression analysis with a significance cut-off of p = 0.05. The p-values for gamma-irradiation and CSC analyses were calculated using a two-tailed t-test method. Statistical analysis for cyclin/Cdk expression was determined by ANOVA.

## Supporting Information

Figure S1 **Drugs ranked by RB status and superimposed for comparison.** (**A**) Sequoia library (1 µM, 268 drugs), (**B**) Spectrum library (1 µM, 2000 drugs), and (**C**) Prestwick library (0.8 µM, 1185 drugs).
(TIF)

Figure S2 **Effect of RB1 status on dose-response curves for 14 human derived TNBC lines treated with PRIMA-1^met^ or fludarabine.** (**A**) Response for each individual line. Values represent the average of 3–5 assays, each performed in triplicate. (**B**) Average response based on RB1 status. RB^+^ lines: MDA-MB-231, HCC38, Hs578t, MDA-MB-157, HCC1954, HCC1569, HCC3153, SUM149 and HCC70. RB^−^ lines: MDA-MB-436, MDA-MB-468, Bt549, Du4475 and HCC1937. PRIMA-1^met^, p = 0.9347. fludarabine, p = 0.6875. (**C**) Average response for hyper- and hypo-phosphorylated pRb states. fludarabine, p = 0.2484. PRIMA-1^met^, p = 0.9884. (**D**) Average response for BaA and BaB subtypes. fludarabine, p = 0.1748. PRIMA-1^met^, p = 0.8237. p-values calculated using nonlinear regression analysis.
(TIF)
