# Supplementary figures and images for: RB1 Status in Triple Negative Breast Cancer Cells Dictates Response to Radiation Treatment and Selective Therapeutic Drugs
Source: PLoS One. 2013 Nov 12;8(11):e78641. doi: 10.1371/journal.pone.0078641 (PMC3827056; doi:10.1371/journal.pone.0078641)

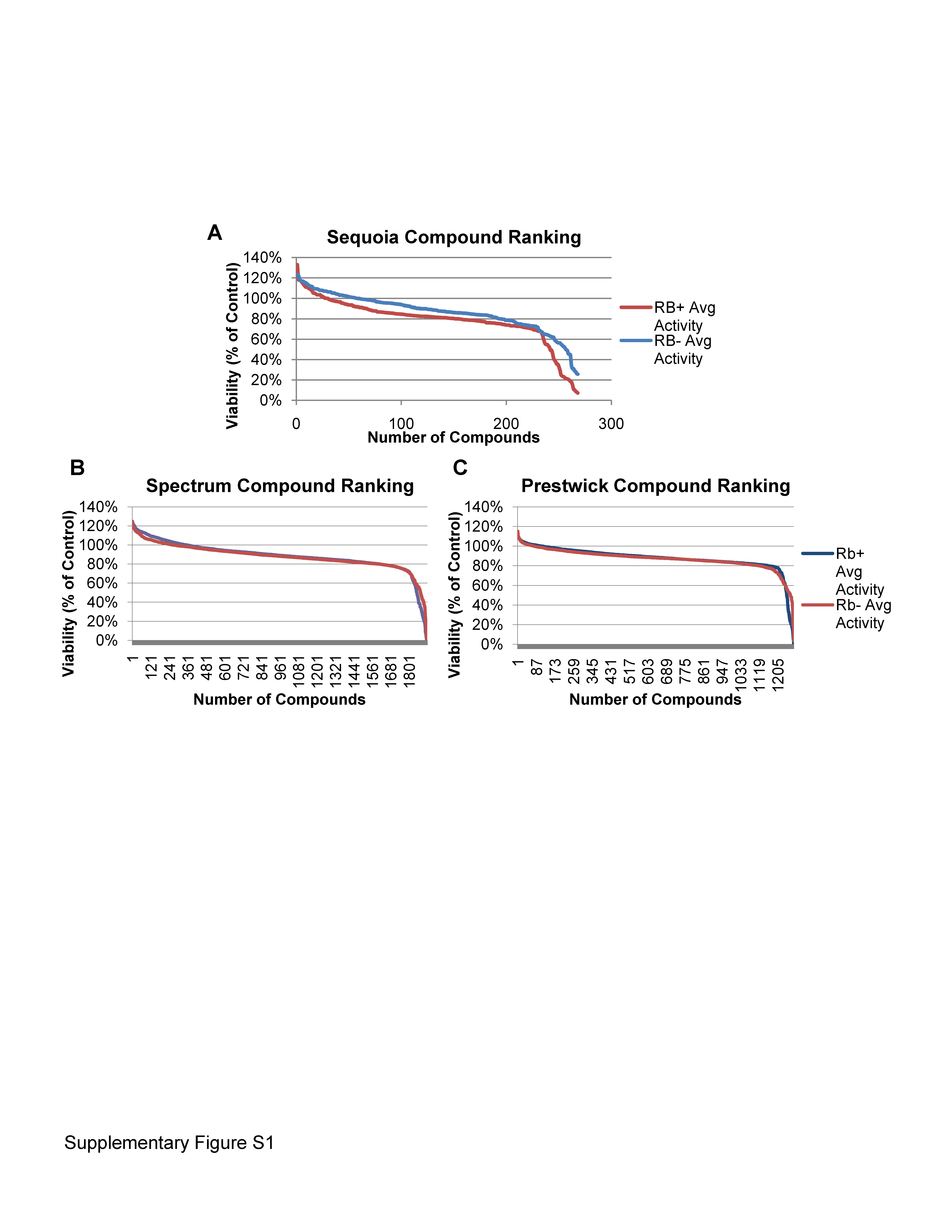

Supplement: Figure S1 — Drugs ranked by RB status and superimposed for comparison. (A) Sequoia library (1 µM, 268 drugs), (B) Spectrum library (1 µM, 2000 drugs), and (C) Prestwick library (0.8 µM, 1185 drugs). (TIF) [file pone.0078641.s001.tif]

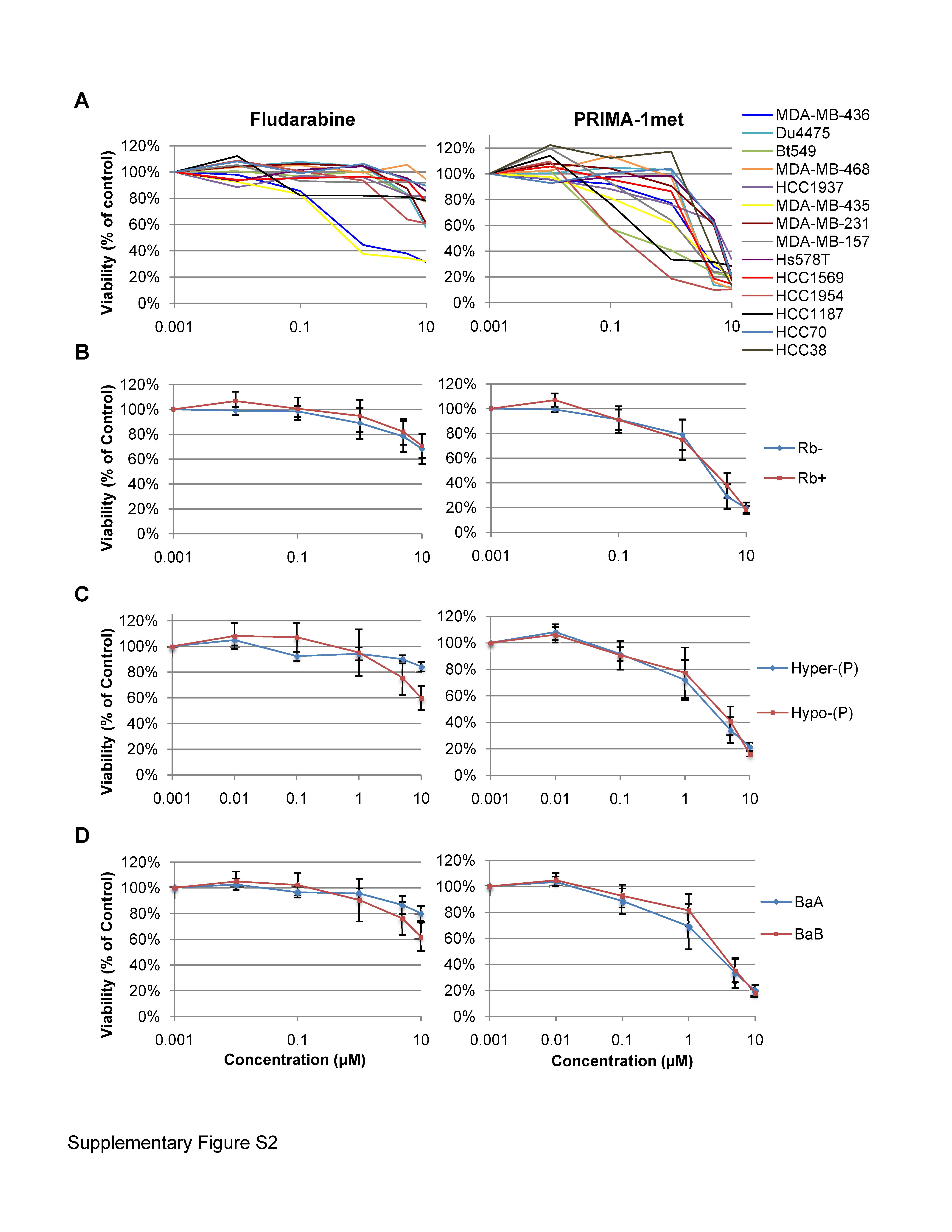

Supplement: Figure S2 — Effect of RB1 status on dose-response curves for 14 human derived TNBC lines treated with PRIMA-1met or fludarabine. (A) Response for each individual line. Values represent the average of 3–5 assays, each performed in triplicate. (B) Average response based on RB1 status. RB+ lines: MDA-MB-231, HCC38, Hs578t, MDA-MB-157, HCC1954, HCC1569, HCC3153, SUM149 and HCC70. RB− lines: MDA-MB-436, MDA-MB-468, Bt549, Du4475 and HCC1937. PRIMA-1met, p = 0.9347. fludarabine, p = 0.6875. (C) Average response for hyper- and hypo-phosphorylated pRb states. fludarabine, p = 0.2484. PRIMA-1met, p = 0.9884. (D) Average response for BaA and BaB subtypes. fludarabine, p = 0.1748. PRIMA-1met, p = 0.8237. p-values calculated using nonlinear regression analysis. (TIF) [file pone.0078641.s002.tif]
